# Nanodevices for the Efficient Codelivery of CRISPR-Cas9 Editing Machinery and an Entrapped Cargo: A Proposal for Dual Anti-Inflammatory Therapy

**DOI:** 10.3390/pharmaceutics14071495

**Published:** 2022-07-19

**Authors:** Alba García-Fernández, Gema Vivo-Llorca, Mónica Sancho, Alicia Belén García-Jareño, Laura Ramírez-Jiménez, Eloísa Barber-Cano, José Ramón Murguía, Mar Orzáez, Félix Sancenón, Ramón Martínez-Máñez

**Affiliations:** 1 Instituto Interuniversitario de Investigación de Reconocimiento Molecular y Desarrollo Tecnológico (IDM), Universitat Politècnica de València, Universitat de València, 46022 Valencia, Spain; gevillo1@posgrado.upv.es (G.V.-L.); muribajo@upvnet.upv.es (J.R.M.); fsanceno@upvnet.upv.es (F.S.); 2 CIBER de Bioingeniería, Biomateriales y Nanomedicina (CIBER-BBN), 28029 Madrid, Spain; 3 Unidad Mixta UPV-CIPF de Investigación en Mecanismos de Enfermedades y Nanomedicina, Centro de Investigación Príncipe Felipe, Universitat Politècnica de València, 46012 Valencia, Spain; msancho@cipf.es (M.S.); agarcia@cipf.es (A.B.G.-J.); 4 Centro de Investigación Príncipe Felipe, 46012 Valencia, Spain; lramirez@cipf.es (L.R.-J.); ebarber@cipf.es (E.B.-C.); 5 Unidad Mixta de Investigación en Nanomedicina y Sensores, UPV-IIS La Fe, 46026 Valencia, Spain

**Keywords:** mesoporous silica nanoparticles, CRISPR-Cas9, gene editing, inflammation, drug delivery

## Abstract

In this article, we report one of the few examples of nanoparticles capable of simultaneously delivering CRISPR-Cas9 gene-editing machinery and releasing drugs for one-shot treatments. Considering the complexity of inflammation in diseases, the synergistic effect of nanoparticles for gene-editing/drug therapy is evaluated in an in vitro inflammatory model as proof of concept. Mesoporous silica nanoparticles (MSNs), able to deliver the CRISPR/Cas9 machinery to edit gasdermin D (GSDMD), a key protein involved in inflammatory cell death, and the anti-inflammatory drug VX-765 (^GSDMD45^CRISPR-VX-MSNs), were prepared. Nanoparticles allow high cargo loading and CRISPR-Cas9 plasmid protection and, thus, achieve the controlled codelivery of CRISPR-Cas9 and the drug in cells. Nanoparticles exhibit GSDMD gene editing by downregulating inflammatory cell death and achieving a combined effect on decreasing the inflammatory response by the codelivery of VX-765. Taken together, our results show the potential of MSNs as a versatile platform by allowing multiple combinations for gene editing and drug therapy to prepare advanced nanodevices to meet possible biomedical needs.

## 1. Introduction

The discovery in bacteria of rudimentary immune systems encoded by genes in proximity to clustered, regularly interspaced short palindromic repeats (CRISPR) and RNA-directed DNA endonucleases, such as CRISP-associated protein 9 (Cas9), recently led to the development of the CRISPR/Cas9 technology to edit a defined sequence in the genome [1,2]. This genome-editing system is formed by three main components: the endonuclease (Cas9), CRISPR (cr)RNA and transactivating CRISPR (tracr)RNA. crRNA results in guidance to the genomic locus of interest and tracrRNA activates endonuclease Cas9 activity by producing targeted double-stranded breaks in chromosomes that can be repaired by either nonhomologous end joining or homologous recombination [3]. For biomedical applications, both crRNA and tracrRNA have been combined in a single guide (sg)RNA. Despite the CRISPR technology’s great potential, the efficient delivery of CRISPR/Cas9 systems to cells remains challenging [4]. Both physical methods and viral vectors have been adopted in the delivery of the Cas9-based gene-editing platform [5]. However, viral vectors are generally concerned about safety issues, whereas most physical methods (e.g., electroporation, microinjection) [6] are applicable only for in vitro delivery due to associated toxicities or poor editing efficiency [7,8]. These limitations have empowered the need to study CRISPR/Cas9 delivery by using nanoparticles [9,10]. Different delivery systems have been developed from lipids [11,12,13], polyribonucleoproteins [14], DNA [15], polymerics [16], virus-like particles [17] and inorganic nanoparticles based on gold nanoparticles [18], mesoporous silica nanoparticles (MSNs) [19], and metal-organic frameworks [20] to deliver the Cas9-based gene-editing system, applied mainly for cancer gene therapy. However, the management of complex diseases by targeting a single molecular pathway has shown limited efficacy considering the complicated mechanisms and signal networks that regulate these diseases [21].

The application of synergistic therapies by combining CRISPR-Cas9 machinery with drugs can be a promising approach to achieve effective therapies [22,23]. However, the codelivery of the CRISPR-Cas9 system and drugs using a nanosystem has been less exploited because very few nanocarriers can efficiently act as a delivery multiplatform. In line with this, MSNs could potentially be exploited to deliver CRISPR-Cas9 gene-editing machinery and an entrapped payload in a one-shot treatment given their unique chemical properties. In the last decade, MSNs, as nanocarriers of cargo molecules, have been extensively explored and applied in the biomedical field from diagnosis to therapy [24,25]. Considering their large surface area and high loading capacity, MSNs can be loaded with a variety of (bio)molecules of different polarities and sizes. MSNs can also be easily functionalized with certain molecular or supramolecular entities to control drug delivery, enhance selectivity to cells and tissues, or increase the escape of the endocytic pathway [26]. The first applied use of MSNs as a dual platform for gene-drug combined therapy was recently described for enhancing cancer therapy. Shao and co-workers developed polyamidoamine-aptamer-coated hollow MSNs to deliver the drug sorafenib and CRISPR/Cas9 to target epidermal growth factor receptor (EGFR) to, thus, enhance hepatocellular carcinoma C treatment [27]. Furthermore, Liu and coworkers developed a virus-like particle, lipid shell-coated MSNs, to enhance immunotherapy in melanoma by the delivery of a small molecule drug (axitinib) and the CRISPR/Cas9 system by targeting programmed death-ligand 1 (PD-L1) [28].

Likewise, we envisioned that MSNs could be excellent candidates for the dual delivery of CRISPR-Cas9 machinery and drugs to enhance current therapies against complex inflammatory disorders. The uncontrolled production of inflammatory mediators and an imbalance of inflammatory cells have been related to acute and chronic inflammatory diseases, such as injury infections, sepsis or arthritis, respiratory disorders, and cancer [29,30]. In most cases, current therapies are still based on the amelioration of inflammatory symptoms and are unable to ensure a complete cure [31,32,33]. Bearing in mind the complexity of inflammation, new efforts still need to be made by blocking different inflammatory pathways. The inflammatory process can be divided into two main mechanisms: activation and release of inflammatory cytokines and activation of lytic cell death referred to as pyroptosis [34]. As previously mentioned, some anti-inflammatory therapies have limited success because they only act by inhibiting the activity of certain cytokines, such as VX-765, but not cell death by pyroptosis [35]. Recently, gasdermin D (GSDMD) has been described as a direct effector of pyroptosis and the deletion of the GSDMD gene with promising results for anti-inflammatory therapy [36].

Accordingly, here we report the first example of MSNs capable of simultaneously delivering the CRISPR-Cas9 technology and an anti-inflammatory drug to improve the efficacy of anti-inflammatory therapies. ^GSDMD45^CRISPR-VX-MSNs, loaded with caspase-1 inhibitor VX-765 (to inhibit caspase-1 activity and pro-inflammatory cytokine release), functionalized with PEI and capped with a CRISPR-Cas9 plasmid with an sgRNA to target the coding region of GSDMD (to inhibit pyroptosis), were prepared. Before this stage, the MSNs coated with a CRISPR-Cas9 plasmid to both edit the GFP gene in U-2 OS-GFP cells and to simultaneously deliver rhodamine B (^GFP38^CRISPR-RhB-MSNs) were used to set up a gene-editing drug-combined system. Nanoparticles allow both high cargo loading and CRISPR-Cas9 plasmid protection to thus achieve the controlled codelivery of CRISPR-Cas9 and the drug in cells. They exhibit GSDMD gene editing by downregulating inflammatory cell death, and also achieve a combined effect on decreasing inflammatory response by the codelivery of VX-765. Taken together, our results show the potential of MSNs as a versatile platform by allowing multiple gene-editing and drug-therapy combinations to prepare advanced nanodevices to meet possible biomedical needs.

## 2. Materials and Methods

### 2.1. General Methods

Powder X-ray diffraction (PXRD), transmission electron microscopy (TEM), scanning electron microscopy (SEM), N_2_ adsorption–desorption isotherms, fluorescence spectrophotometry, Fourier-transform infrared spectroscopy (FTIR), thermogravimetric and elemental analyses were employed for materials characterization. PXRD measurements were taken on a Seifert 3000TT diffractometer by using CuK_α_ radiation. TEM images were acquired under a JEOL TEM-1010 electron microscope working at 100 kV. The N_2_ adsorption–desorption isotherms were recorded in a Micromeritics TriStar II Plus automated analyzer. To determine the zeta potential of the bare and functionalized nanoparticles, Zetasizer Nano ZS equipment (Malvern Instruments, Malvern, UK) was used. Samples were dispersed in distilled water at a concentration of 1 mg/mL. The zeta potential was calculated from the particle mobility values by applying the Smoluchowski model. The average of five recordings was reported as the zeta potential. Measurements were taken in triplicate at 25 °C. Dynamic light scattering (DLS) studies to determine particle size were conducted at 25 °C in a Malvern Zetasizer Nano ZS instrument. All the measurements were taken in triplicate on previously sonicated highly dilute water dispersions. Fluorescence measurements were taken with a JASCO FP-8500 spectrophotometer. FTIR measurements were recorded by a Bruker Tensor 27 spectrometer. The thermogravimetric analyses were carried out by TGA/SDTA 851e Mettler Toledo equipment in an oxidant atmosphere (air, 80 mL/min) with a heating program that consisted of a heating ramp of 10 °C per min from 393 K to 1273 K, and an isothermal heating step at this temperature for 30 min. An elemental analysis was run in a CE Instrument EA-1110 CHN elemental analyzer. Cell viability measurements were taken with a Wallac 1420 workstation. Confocal microscopy imaging was performed with a Leica TCS SP8 HyVolution II (Leica Microsystems Heidelberg GmbH, Heidelberg, Germany) inverted laser scanning confocal microscope.

### 2.2. Preparation of MSNs

CTAB (1.00 g, 2.74 mmol) was dissolved in 480 mL of deionized H_2_O before adding a solution of NaOH (3.5 mL, 2.00 M). The solution temperature was adjusted to 80 °C and then TEOS (5.00 mL, 2.57 × 10^−2^ mol) was added dropwise to the surfactant solution with maximum stirring. The mixture was stirred for 2 h to give a white precipitate. The solid was isolated by centrifugation and washed with deionized H_2_O until a neutral pH was reached. Finally, the solid was dried at 60 °C. To prepare the final porous material, MSNs were calcined at 550 °C in an oxidant atmosphere to remove the template phase.

### 2.3. Preparation of PEI-RhB-MSNs

First, 50 mg of calcined MSNs and 28 mg (0.16 mmol) of rhodamine B (RhB) were suspended in 10 mL of ethanol. The mixture was stirred for 24 h at room temperature to achieve maximum loading in pores. Afterward, the solid was isolated by centrifugation and 25 mg of branched polyethyleneimine (PEI) (M.W. 10,000) (Polysciences) in ethanol were added. The suspension was stirred for 3 h. Finally, the pink solid was isolated, washed with ethanol and dried at 37 °C. Empty nanoparticles were prepared following a similar procedure to obtain a white solid referred to as PEI-MSNs.

### 2.4. Preparation of the CRISPR-Cas9 Vector to GFP Edition

The oligonucleotides encoding sgRNA were designed based on the genomic sequence to edit according to the literature [37,38]. A length of 20 nucleotides complementary to 20 nucleotides of the GFP sequence followed for a protospacer adjacent motif (PAM) essential for cleavage by Cas nuclease: 5′-(N20)-NGG-3′. The CRISPR plasmid pX330-U6-Chimeric_BB-CBh-hSpCas9 (Plasmid #42230) was obtained from Addgene (Teddington, UK). Guide oligonucleotides to edit GFP positions were obtained from Sigma Aldrich (Madrid, Spain). First, plasmid pX330-U6-Chimeric_BB-CBh-hSpCas9 was digested by the BpiI (BbsI) enzyme (Sigma Aldrich, Madrid, Spain) and ligated with the annealed oligonucleotides following standard procedures. The presence of the insert of GFP gRNA was confirmed by sequencing.

### 2.5. Assembly and Characterization of CRISPR-RhB-MSNs

To assess the efficacy of DNA binding to MSNs, agarose gel electrophoresis of the naked plasmid and CRISPR-RhB-MSNs was performed. For this purpose, purified plasmid and PEI-RhB-MSNs at various molar ratios (1:10, 1:25, 1:200, 1:500) were mixed in Opti-MEM and incubated at 37 °C for 30 min. Then, nanoparticles were centrifuged, and supernatants were loaded in agarose gel in order to detect the unbound plasmid. The resulting bands were inspected via ethidium bromide staining.

### 2.6. Preparation of CRISPR Plasmid-Containing PEI-MSNs and PEI-RhB-MSNs

A suspension of PEI-MSNs or PEI-RhB-MSNs (25 µg/mL) was incubated with 1 µg/mL of the corresponding CRISPR-Cas9 vector for GFP editing in Opti-MEM for 30 min. The following vectors, ^GFP38^CRISPR-Cas9, ^GFP149^CRISPR-Cas9, ^GFP178^CRISPR-Cas9, and ^scrambled^CRISPR-Cas9, were used to yield solids ^GFP38^CRISPR-MSNs, ^GFP149^CRISPR-MSNs, ^GFP178^CRISPR-MSNs, and ^scrambled^CRISPR-MSNs, respectively. A similar procedure was performed with the corresponding CRISPR-Cas9 vector for GSDMD editing. The following vectors, ^GSDMD38-^CRISPR-Cas9, ^GSDMD45^CRISPR-Cas9, and ^GSDMD-S^CRISPR-Cas9, were used to obtain solids ^GSDMD38^CRISPR-MSNs, ^GSDMD45^CRISPR-MSNs, and ^GSDMD-S^CRISPR-MSNs, respectively.

### 2.7. Stability Studies of the ^GFP38^CRISPR-Cas9 Vector in MSNs

^GFP38^CRISPR-RhB-MSNs and free ^GFP38^CRISPR-Cas9 DNA were incubated at 37 °C for 10 min with DNAase I enzyme (0.5 ng/mL). Then the DNA bound to nanoparticles was released by using heparin (7.5 mg/mL) and analyzed by agarose electrophoresis. ^GFP38^CRISPR-RhB-MSNs were treated with heparin (7.5 mg/mL) in the absence of DNAse I to confirm the correct DNA disassociation. In contrast, heparin was previously added to disassemble the DNA from ^GFP38^CRISPR-RhB-MSNs, and DNAse I was added to corroborate the protection of the vector on MSNs.

### 2.8. Preparation of ^GFP38^CRISPR-RhB*-MSNs

First, 2 mg of rhodamine B isothiocyanate (RBIT) were added with 20 µL of (3-aminopropyl)triethoxysilane (APTES) in 2 mL of anhydrous ethanol. The mixture was stirred in the dark at room temperature overnight. Then, 10 mg of MSNs were dispersed in 5 mL of anhydrous ethanol and 40 µL of the RBIT/APTES mixture were added. The suspension was left in the dark for 5.5 h at room temperature. Nanoparticles were washed and dried to yield RhB*-MSNs. The PEI-RhB*-MSNs and CRISPR-RhB*-MSNs were obtained following the synthetic procedure described in Section 2.3 and Section 2.6.

### 2.9. Delivery Studies from ^GFP38^CRISPR-RhB-MSNs

First, 1 mg of solid ^GFP38^CRISPR-RhB-MSNs was suspended in 1 mL of simulated plasma at pH 7.0 or in 1 mL of simulated plasma at pH 5.0 [36]. Suspensions were stirred at 37 °C. At scheduled times (0, 15, 30, 60, and 90 min), an aliquot was obtained from each suspension and centrifuged to eliminate the solid. Rhodamine B delivery was followed by measuring fluorescence emission at 585 nm (λ_ex_ = 525 nm).

### 2.10. Preparation of PEI-VX-MSNs

A saturated solution of VX-765 (Selleckchem, S2228) (15 mg in 1 mL of ethanol) was prepared. The solution was separated into two aliquots and added to two different cycles to calcined MSNs. MSNs (30 mg) were extended in a plate and the first VX-765 aliquot was added dropwise. Then, the mixture was dried at 37 °C and the second impregnation step was carried out. The material was finally dried at 37 °C. Next, the solids suspended in 4 mL of ethanol and 15 mg of PEI suspension in 1 mL of ethanol were added. The suspension was stirred for 3 h. Finally, the white solid was isolated, washed with ethanol, and dried at 37 °C.

### 2.11. Preparation of CRISPR Plasmid-Containing PEI-VX-MSNs

To obtain the CRISPR-VX-MSNs, 1 µg/mL of the corresponding plasmid was incubated for 30 min with PEI-VX-MSNs in Opti-MEM (25 µg/mL) at room temperature. Commercial pCMV-Cas9-GFP (CAS9GFPP) to edit GSDMD at gene positions 37 and 45 were obtained from Sigma Aldrich. The mixture was prepared by using vectors ^GSDMD38^CRISPR-Cas9, ^GSDMD45^CRISPR-Cas9, and ^GSDMD-S^CRISPR-Cas9 to yield solids ^GSDMD38^CRISPR-VX-MSNs, ^GSDMD45^CRISPR-VX-MSNs, and ^GSDMD-S^CRISPRRhB-MSNs, respectively.

### 2.12. Cell Culture

The human osteosarcoma U-2 OS-GFP cells were a kind gift from Susana Llanos from the Centro Nacional de Investigaciones Oncológicas (CNIO, Madrid, Spain). Cells were maintained in DMEM (Gibco, Madrid, Spain), supplemented with 10% FBS (Sigma Aldrich), and incubated in 20% O_2_ and 5% CO_2_ at 37 °C. The human leukemia monocytic THP-1 cells were obtained from ATCC. Cells were maintained in RPMI (Gibco), supplemented with 10% FBS, and incubated in 20% O_2_ and 5% CO_2_ at 37 °C.

### 2.13. Toxicity Studies

The U-2 OS-GFP cells were seeded in a 24-well plate at a cellular density of 25,000 cells/well and treated with different concentrations of ^GFP38^CRISPR-RhB-MSNs (0, 25, 50, and 100 µg/mL). Cells were incubated for 24 h and 48 h, and viability was determined by adding cell proliferation reagent WST-1 (Sigma Aldrich) according to manufacturer’s instructions. After 1 h of incubation, absorbance at 595 nm was measured in a Wallac 1420 workstation.

THP-1 cells were seeded in a p96-well plate at a cellular density of 10,000 cells/well and treated with different concentrations of ^GSDMD45^CRISPR-RhB-MSNs (0, 25, 50, and 100 µg/mL). Cells were incubated for 24 and 48 h, and then cell viability was determined by the WST-1 assay and inflammatory cell death by LDH activity assay (Promega, G7891, Madrid, Spain). For LDH activity, cell supernatants were analyzed by following the manufacturer’s instructions. As a positive control, 1% Triton-X100 was added.

### 2.14. Cellular Uptake Studies

The U-2 OS cells were seeded in a 6-well plate and treated with 25 µg/mL of ^GFP38^CRISPR-RhB*-MSNs (containing covalently anchored rhodamine B) nanoparticles for 15, 30, 60, and 120 min. Cells were washed with PBS to remove the non-internalized nanoparticles and collected for rhodamine B quantification by flow cytometry in a CytoFLEX S (Beckman-Coulter, Brea, CA, USA) cytometer and analyzed by the CytExpert 2.3 software. To demonstrate endosomal escape, the U-2 OS cells were seeded on glass coverslips in 6-well plates and incubated at 37 °C for 24 h. Then, 25 µg/mL of the ^GFP38^CRISPR-RhB*-MSNs were added, and cells were incubated in the presence of a green lysotracker (LysoTracker Green DND-26 from Thermo Fisher, L7256, Madrid, Spain) or an endosomal marker (CellLight Late Endosomes-GFP, BacMam 2.0 from Thermo Fisher, C10588) for 1 h. Then coverslips were washed with PBS and new media were added with DNA marker Hoechst 33342 (Thermo Fisher, 10150888) and cell membrane marker Wheat Germ Agglutinin, Alexa Fluor™ 647 Conjugate (Invitrogen, W32466, Madrid, Spain) at a final concentration of 2 µg/mL and 5 µg/mL, respectively. Living cells were visualized in real time with confocal microscope Leica TCS SP8 HyVolution II equipped with CO_2_ and temperature control and a resonant scanner for live-cells studies. Moreover, treatment with the endocytic inhibitor Dynasore (Sigma-Aldrich, 324410) at a final concentration of 100 µM was included as a to cellular uptake control.

### 2.15. Gene Editing of GFP in U-2 OS-GFP Cells

Cells were cultured in 6-well plates at 250,000 cells/well for 24 h before transfection. Then, cells were incubated with the nanoparticles ^GFP38^CRISPR-MSNs, ^GFP149^CRISPR-MSNs, ^GFP178^CRISPR-MSNs, and ^scrambled^CRISPR-MSNs at 25 µg/mL for 4 h, and the media was replaced. After 48 h, cells were washed with PBS and the DNA marker Hoechst 33342 was added. Living cells were visualized in a confocal Leica TCS SP8 HyVolution II microscope. The quantification of GFP-associated fluorescence intensity for the different treatments was performed by analyzing the confocal images with the Image J software. The expression of the GFP levels in the U-2 OS-GFP cells was confirmed by Western blot analysis. Whole-cell extracts were obtained by lysing cells in a buffer containing 25 mM Tris-HCl, pH 7.4, 1 mM EDTA, 1 mM EGTA, and 1% SDS, plus protease and phosphatase inhibitors. The cells were mixed with the buffer by using a micropipette for a few minutes (2–3 min). The suspension was centrifuged at 14,000 rpm for 10 min, the supernatants were collected in new tubes, and the protein concentration was determined by using the Pierce BCA Protein Assay Kit (Thermo Fisher, 10741395). Lysates were resolved by SDS-PAGE, transferred to nitrocellulose membranes, blocked with 5% non-fat milk, washed with 0.1% Tween/PBS, and incubated overnight with a specific primary antibody. Primary and secondary antibodies were prepared in 5% non-fat milk. GFP (Santa Cruz Biotechnology, sc-9996, Heidelberg, Germany) antibody was prepared at 1:1000 dilution, and α-Tubulin (YL1/2) antibody (Abcam, ab6160, Cambridge, UK) at 1:3000 dilutions. Membranes were washed and incubated with the correspondent secondary antibody conjugated with horseradish peroxidase, anti-mouse IgG antibody (Abcam, ab205719), and anti-rat IgG antibody (Abcam, ab182931), respectively, and prepared at 1:3000 dilutions. Chemiluminescence was detected after Lumi-Light Western Blotting Substrate (Roche) incubation.

### 2.16. Gene Editing of GFP and Cargo Co-Delivery in the U-2 OS-GFP Cells

Cells were cultured in 6-well plates at 250,000 cells/well 1 day before transfection. Then, cells were incubated with the prepared ^GFP38^CRISPR-RhB-MSNs, ^GFP149^CRISPR-RhB-MSNs, ^GFP178^CRISPR-RhB-MSNs, and ^scrambled^CRISPR-RhB-MSNs at 25 µg/mL for 4 h, and media were replaced. After 48 h, cells were washed with PBS and DNA marker Hoechst 33342 was added. Slides were visualized under a confocal microscope Leica TCS SP8 HyVolution II with the Leica Application Suite X (LAS X) software. The quantification of the GFP-associated fluorescence intensity and rhodamine B-associated fluorescence intensity for the different treatments was performed by analyzing confocal images with the Image J 1.53K software developed at the National Institutes of Health and the Laboratory for Optical and Computational Instrumentation (LOCI, University of Wisconsin) by Tiago Ferreira and Wayne Rasband. GFP fluorescence was also quantified by the HCS system IN Cell Analyzer 2200.

### 2.17. Cellular Uptake Studies with ^GSDMD45^CRISPR-RhB-MSNs in THP-1 Cells

In a first step, ^GSDMD45^CRISPR-RhB-MSNs uptake was analyzed by flow cytometry in the THP-1 cells. For this purpose, the THP-1 cells were seeded in a 6-well plate at 800,000 cells/well in RPMI supplemented with 1% FBS and treated with 25 µg/mL of nanoparticles for 15, 30, 60, and 120 min. Cells were washed with PBS to remove the non-internalized nanoparticles and collected for rhodamine B quantification by flow cytometry. The single-cell fluorescence was analyzed by using CytoFLEX S (Beckman-Coulter, USA) and analyzed with the CytExpert 2.3 software. In addition, internalization of the nanoparticles in THP-1 cells was followed by confocal microscopy. Cells were seeded in 6-well plates and treated with 25 µg/mL of ^GSDMD45^CRISPR-RhB-MSNs for 24 h. Finally, cells were washed with PBS and Hoechst 33342 was added at 2 µg/mL. Living cells were visualized in a confocal Leica TCS SP8 HyVolution II microscope. CRISPR-Cas9 delivery using MSNs was analyzed in the THP-1 cells. For this purpose, the ^GSDMD45^CRISPR-Cas9 vector was labeled with Cy7 following the manufacturer’s instructions from Label IT Tracker Intracellular Nucleic Acid Localization Kit (Mirus), and the nanoparticles *^GSDMD45^CRISPR-MSNs were prepared and added to the THP-1 cells for 24 h. Finally, cells were washed with PBS and treated with early green lysotracker and the DNA marker Hoechst 33342.

### 2.18. GSDMD Gene Editing in the THP-1 Cells

THP-1 cells were seeded in a 6-well plate at 800,000 cells/well in RPMI supplemented with 1% FBS. Cells were incubated with the prepared ^GSDMD-S^CRISRP-MSNs, ^GSDMD38^CRISRP-MSNs, and ^GSDMD45^CRISRP-MSNs at 25 µg/mL for 48 h. Then cells were washed and collected to analyze the GSDMD levels in cell lysates by Western blot. To determine the amount of GSDMD, whole-cell extracts were obtained as described previously in Section 2.15. The primary antibody against GSDMD (Sigma-Aldrich, SAB1411444) and GAPDH antibody (Fisher, 11335232) was used at a 1:1000 dilution. Membranes were washed and probed with the appropriate secondary antibody conjugated with horseradish peroxidase, anti-rabbit IgG antibody (Abcam, ab205718) and anti-mouse IgG antibody (Abcam, ab205719), respectively, for enhanced chemiluminescence detection.

### 2.19. Evaluation of Dual Therapy by Using ^GSDMD^CRSIRP-VX-MSNs

The THP-1 cells were seeded in a 6-well plate at 800,000 cells/well in RPMI supplemented with 1% FBS. Cells were incubated with ^GSDMD-S^CRISRP-MSNs, ^GSDMD38^CRISRP-MSNs, ^GSDMD45^CRISRP-MSNs, ^GSDMD-S^CRISRP-VX-MSNs, ^GSDMD38^CRISRP-VX-MSNs, and ^GSDMD45^CRISRP-VX-MSNs at 25 µg/mL for 36 h. VX-765 (1 µm) was added as a control. After 36 h of incubation, the THP-1 cells were stimulated by using lipopolysaccharide (LPS) from *Escherichia coli* O111:B4 (Sigma Aldrich, L2630-100MG) at 100 ng/mL for 12 h and nigericin (NG) (Sigma Aldrich, N7143-5MG) at 10 µM for 30 min. Finally, cells were collected LDH activity and IL-1β levels were analyzed in cell supernatants. IL-1β levels were measured by enzyme-linked immunosorbent assay (ELISA) (BD Biosciences Ref 557953, Madrid, Spain) according to the manufacturer’s instructions.

## 3. Results

### 3.1. Synthesis and Characterization of CRISPR-MSNs

**Figure 1:**
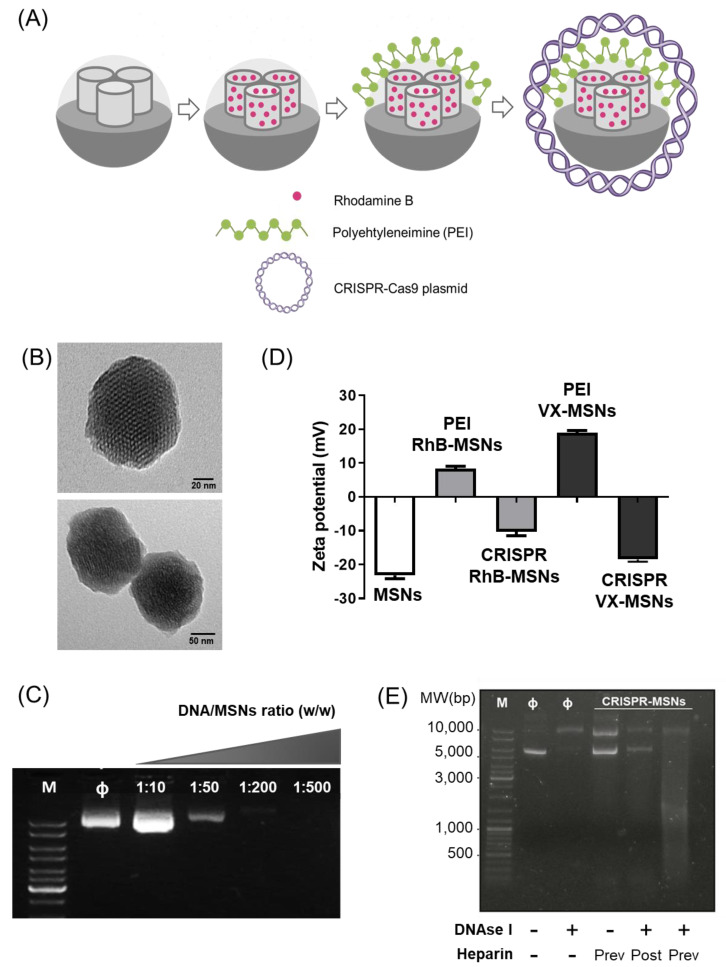
(**A**) Schematic representation of CRISPR-MSNs synthesis. (**B**) TEM images of MSNs (top) and PEI-RhB-MSNs (bottom). (**C**) The gel shift mobility assay of ^GFP38^CRISPR-RhB-MSN generated at different DNA/PEI-MSNs ratios. M, MW marker; Φ, naked DNA plasmid (CRISPR-Cas9) as a negative control. (**D**) The zeta potential of MSNs, PEI-RhB-MSNs and ^GFP38^CRISPR-RhB-MSNs. (**E**) Stability studies of ^GFP38^CRISPR-MSN in the presence of DNAase I. Lane 1, MW marker (M). Lane 2, the naked GFP38CRISPR plasmid (Φ). Lane 3, ^GFP38^CRISPR treated with DNAse I. Lane 4, ^GFP38^CRISPR-RhB-MSNs treated with heparin (Prev) to disassemble the MSNs-DNA complex. Lane 5, the ^GFP38^CRISPR-RhB-MSNs complex treated with DNAse I and then with heparin (post). Lane 6, ^GFP38^CRISPR-RhB-MSNs were previously disassembled with heparin (prev) and finally treated with DNAse I. All the experiments represent at least three independent experiments.

MSNs were selected as scaffolds to prepare CRISPR-MSNs for the co-delivery of the gene-editing machinery and drugs considering their unique chemical properties of large surface area and high loading capacity, as well as the easy functionalization of their external surfaces with molecules acting as gatekeepers to control cargo delivery and to thus enhance selectivity to cells and tissues. In a first attempt, the pores of nanoparticles were loaded with fluorescent dye RhB (as model cargo) before being capped with a polyethyleneimine (PEI) layer (PEI-RhB-MSNs) via the electrostatic interactions between the negatively charged external surface of nanoparticles and the positively charged PEI polymer. The PEI cationic polymeric layer was used as (i) a suitable positive layer to attach the negatively charged CRISPR-Cas9 vector, and (ii) to enable nanoparticles for endosomal escape, which is required for enhanced plasmid delivery to the cytosol. Finally, the CRISPR-Cas9 vector (editing the GFP38 gene position) was adsorbed onto PEI-RhB-MSNs to produce the final nanoparticles ^GFP38^CRISPR-RhB-MSNs (Figure 1A). The CRISPR-Cas9 vector included both single-guide RNA (sgRNA) and endonuclease Cas9 in one autonomously replicable plasmid (Figure S1). Similar nanoparticles containing a CRISPR-Cas9 vector to target the coding region of GSDMD and loaded with the drug VX-765 were also synthesized (^GSDMD45^CRISPR-VX-MSNs). For both systems, unloaded nanoparticles were also prepared (see below).

Materials were characterized by standard procedures (Figure S2). The TEM images of MSNs as-made and PEI-RhB-MSNs showed mesoporous spherical nanoparticles whose average size was ca. 100 nm, which is suitable for intracellular delivery [39,40,41] (Figure 1B). After characterizing the starting material, different DNA/PEI-RhB-MSNs (*w*/*w*) ratios were tested to assess the nucleic acid binding capacity of the nanoparticle. For this purpose, the CRISPR-Cas9-free plasmid was incubated with PEI-RhB-MSNs, and the obtained nanoparticles were subjected to an electrophoretic mobility shift assay. An optimal 1:25 vector/PEI-RhB-MSNs ratio was selected between the studied ranges to obtain the final nanodevice (Figure 1C). The proper assembly of nanoparticles was also confirmed by the zeta potential (Figure 1D), which was negative for the starting MSNs (−24 ± 1 mV) and became positive after loading (with RhB or VX-765) and PEI coating (+7.48 ± 0.50 and 19 ± 3 mV for PEI-RhB-MSNs and PEI-VX-MSNs, respectively) and returned to negative values after plasmid adsorption (−12 ± 2 and −19 ± 10 mV for ^GFP38^CRISPR-RhB-MSNs and ^GSDMD45^CRISPR-VX-MSNs, respectively). In addition, studies in the presence of DNAase I verified an improvement in ^GFP38^CRISPR-Cas9 plasmid stability after its adsorption to the external surface of the PEI-coated MSNs. As seen in Figure 1E, the ^GFP38^CRISPR-Cas9-free plasmid treated with DNAse I had completely degraded (Figure 1E, lane 3), whereas the CRISPR-Cas9 vector in nanoparticles was protected from DNAase I digestion under conditions in which the free plasmid was unstable (Figure 1E, lanes 5). In lane 4, the proper disassembly of the CRISPR-Cas9 vector from MSNs is observed upon heparin addition. The digestion of the DNA disassembled from ^GFP38^CRISPR-RhB-MSNs was observed after heparin and DNAse I treatment (Figure 1E, lane 6).

### 3.2. CRISPR-MSNs Efficiently Deliver Their Cargo and CRISPR-Cas9 Machinery in Cells

**Figure 2:**
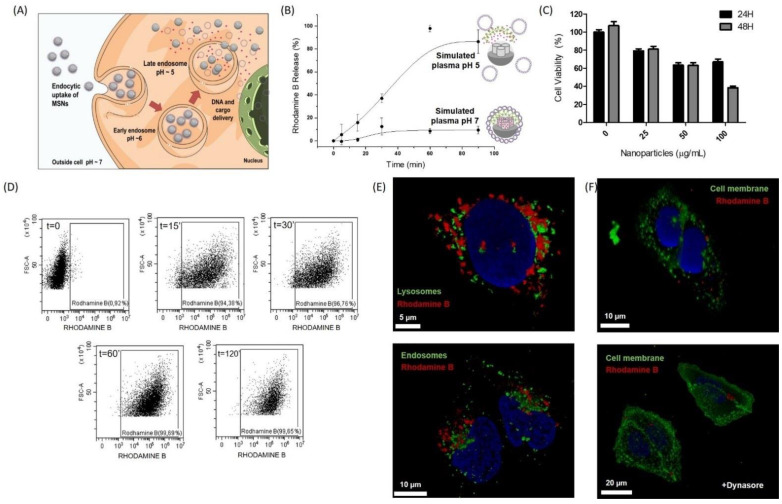
Internalization and delivery characterization of CRISPR-MSNs. (**A**) Scheme of CRISPR and dye cell delivery by CRISPR-RhB-MSNs. Figure 1B was produced by using a template from the Server Medical Art platform. (**B**) Delivery profile of ^GFP38^CRISPR-RhB-MSNs in the presence of simulated plasma at pH 7 or pH 5. Data represent the mean ± SEM of at least three independent experiments. (**C**) Cell viability studies by WST-1 assays at different ^GFP38^CRISPR-MSNs concentrations at 24 (black bars) and 48 h (grey bars). Data represent the mean ± SEM of at least three independent experiments. (**D**) Cellular internalization of ^GFP38^CRISRP-RhB*-MSNs in the U-2 OS cells at different times measured by flow cytometry. (**E**) Cellular uptake of ^GFP38^CRISRP-RhB*-MSNs in the presence of the endosomal marker (CellLight Late Endosomes-GFP, BacMam 2.0) (green) after 30 min of incubation. ^GFP38^CRISRP-RhB*-MSNs (red) in the U-2 OS cells with the lysosomes maker (green) (LysoTracker Green DND-26) or after 1 h of incubation. Representative images of the confocal experiments in at least three independent experiments. (**F**) Cellular internalization of ^GFP38^CRISRP-RhB*-MSNs (red) in the U-2 OS cells stained with the cell membrane marker (green) in the absence (top) or presence (bottom) of endocytic inhibitor dynasore. Representative images of the confocal experiments in at least three independent experiments.

To achieve proper CRISPR-Cas9 and cargo delivery, nanoparticles were designed after taking into account that nanoparticles are internalized by endocytosis. Then nanoparticles move through endosomal compartments. When they reach late endosomes, in which the pH is mildly acidic, PEI coating acts as a proton sponge. This effect results in the disruption of endosomes to thus allow endosomal escape, proper plasmid delivery, and controlled cargo delivery (Figure 2A). The cargo release studies conducted with ^GFP38^CRISPR-RhB-MSNs in simulated plasma at pH 7 or by mimicking the endosomal compartment from cells at pH 5 demonstrated that cargo delivery was poor at pH 7, whereas marked RhB delivery was found at acidic pH 5 (Figure 2B). Cargo release was attributed to the partial protonation of the PEI coating, which induced its disassembly from MSNs. Biocompatibility studies demonstrated that ^GFP38^CRISPR-RhB-MSNs were well-tolerated by the U-2 OS-GFP cells at concentrations of 25 µg mL^−1^ after 48 h of incubation (Figure 2C). Cellular uptake efficiency was also studied by flow cytometry by using similar nanoparticles, but containing covalently anchored RhB (i.e., ^GFP38^CRISPR-RhB*-MSNs) (Figure 2D). The kinetic studies indicated that 90% of the cellular population incorporated nanoparticles in 15 min. Moreover, to demonstrate the endosomal escape of nanoparticles, a key point to achieve plasmid delivery to the cytosol, the U-2 OS cells were treated with the RhB-labeled nanoparticles (i.e., ^GFP38^CRISPR-RhB*-MSNs) in the presence of a lysosomal marker (LysoTracker Green DND-26) (Figure 2E, up) or an endosomal marker (CellLight Late Endosomes-GFP, BacMam 2.0) (Figure 2E, down). The fluorescence confocal microscopy analysis showed no overlapping signals between lysosomes (green) and nanoparticles (red) after 1 h of transfection, which confirmed the endosomal escape of nanoparticles. Besides, the cellular uptake in the presence of endocytic inhibitor dynasore significantly reduced, which confirmed endocytosis as an internalization mechanism (Figure 2F). 

### 3.3. MSNs as a Versatile System for the Codelivery of Drugs and the Gene-Editing CRISPR-Cas9 Machinery

**Figure 3:**
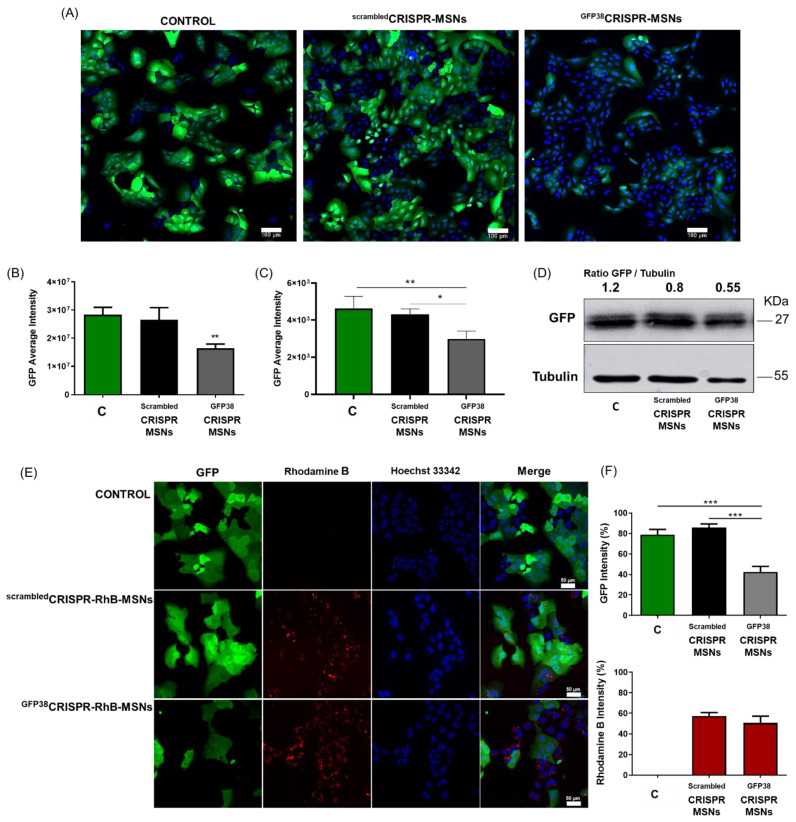
Dual gene editing and cargo delivery in the U-2 OS-GFP cells. (**A**) Confocal microscopy images of genome editing by the CRISPR-Cas9 system delivered by MSNs as nanocarriers. Transfection efficiency is judged by fluorescent intensity and the proportion of cells in the population showing GFP expression. Green depicts the GFP cells and blue marks the nucleus with Hoechst 33342. The scale bar represents 10 µm. Representative images of the confocal experiments in at least three independent experiments. (**B**) GFP quantification by the confocal images analysis by using the Image J software. Data represent the mean ± SEM of at least three independent experiments. The control cells (untreated) are referred to as C. A statistical analysis was performed by applying a one-way ANOVA with multiple comparisons (* *p* < 0.05, ** *p* < 0.025, *** *p* < 0.001). (**C**) GFP quantification by IN Cell Analyzer 2200. Data represent the mean ± SEM of at least three independent experiments. A statistical analysis was performed by applying a one-way ANOVA with multiple comparisons (* *p* < 0.05, ** *p* < 0.025, *** *p* < 0.001). (**D**) Western blot analysis and quantification of the GFP levels expressed in the cell lysates of the CRISPR-MSNs editing studies. Representative images of at least three independent experiments. (**E**) Confocal microscopy images of genome editing and cargo delivery. Transfection efficiency is judged by fluorescent intensity and the proportion of cells in the population showing GFP (green) expression and delivery efficiency by the fluorescent intensity of Rhodamine B (red) and the nucleus in blue marked with Hoechst 33342. The scale bar represents 10 µm. Representative images of the confocal experiments in at least three independent experiments. (**F**) GFP (up) and Rhodamine B (down) quantification by the confocal images analysis by using the Image J software. The untreated cells, the negative control, are referred to as C. Data represent the mean ± SEM of at least three independent experiments. A statistical analysis was performed by applying a one-way ANOVA with multiple comparisons (* *p* < 0.05, ** *p* < 0.025, *** *p* < 0.001).

To assess the efficiency of CRISPR-Cas9 delivery and subsequent gene editing, we targeted the coding region of GFP in the U-2 OS-GFP cells. Gene editing was expected to result in GFP gene expression loss and diminished cellular green fluorescence. The confocal microscopy analysis of the U-2 OS-GFP cells treated with unloaded ^GFP38^CRISPR-MSNs revealed a remarkable decrease in green fluorescence intensity (Figure 3A,B). The diminished GFP expression was also quantified by cytomics by using IN Cell Analyzer 2200 (Figure 3C), as confirmed by a Western blot analysis (Figure 3D). In contrast, similar nanoparticles containing a scrambled plasmid (^scrambled^CRISPR-MSNs) displayed no changes in either fluorescence or GFP expression. Finally, similar studies were carried out by using ^GFP38^CRISPR-RhB-MSNs to demonstrate that particles simultaneously delivered the plasmid and the entrapped cargo. The confocal microscopy analysis showed in the ^GFP38^CRISPR-RhB-MSNs-treated U-2 OS-GFP cells that the GFP expression levels lowered and RhB fluorescence was observed (Figure 3E). Distinct sgRNAs targeting other GFP gene positions were also cloned, and the corresponding nanoparticles ^GFP149^CRISPR-MSNs, ^GFP149^CRISPR-RhB-MSNs, ^GFP178^CRISPR-MSNs, and ^GFP178^CRISPR-RhB-MSNs were tested in the U-2 OS-GFP cells (Figures S1, S3 and S4) with similar results.

### 3.4. MSNs for the Simultaneously Delivery of Gene-Editing Machinery and Drugs in Inflammatory Diseases

Encouraged by these results, we moved one step forward and used nanoparticles to simultaneously deliver a drug and the CRISPR-Cas9 plasmid as a double hit strategy for inflammatory treatment. As previously discussed, to solve complex inflammation, improved strategies are needed to achieve a complete therapeutic effect. We evaluated if the proposed dual therapy with the prepared ^GSDMD45^CRISPR-VX-MSNs nanoparticles, loaded with caspase-1 inhibitor VX-765 and capped with a CRISPR-Cas9 plasmid to target the coding region of GSDMD, could be a potential approach for controlling the dysregulated inflammatory response. We first assessed the possible toxicity of the prepared nanodevice in the monocytes THP-1 cells, selected as a cellular model to evaluate the inflammatory response and therapy. The cell viability studies demonstrated that ^GSDMD45^CRISPR-MSNs were well-tolerated by the monocytes THP-1 cells at 25 µg mL^−1^ after 48 h (Figure S5).

**Figure 4:**
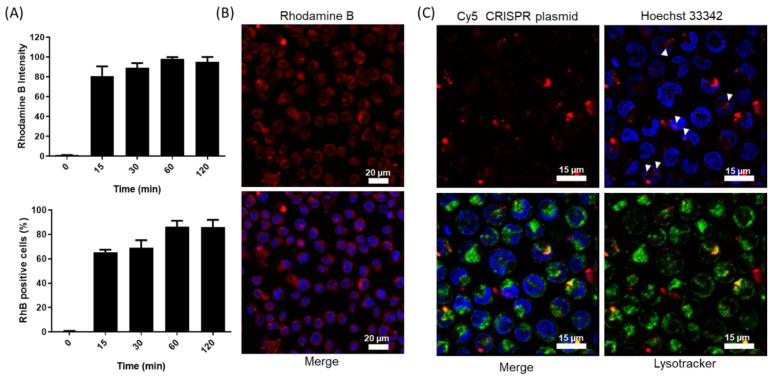
CRISPR-MSNs dual delivery in the THP-1 cells. (**A**) Flow cytometry studies in THP-1 cells by using ^GSDMD45^CRISPR-RhB-MSNs. Rhodamine B intensity (top) and Rhodamine B positive cells (bottom). Data represent the mean ± SEM of at least three independent experiments. (**B**) Cargo release from ^GSDMD45^CRISPR-RhB-MSNs in the THP-1 cells after 24 h. Confocal images showing Rhodamine B released from nanoparticles (top) and merged channels (bottom) with the cells stained with nuclei marker Hoechst 33342 (in blue). (**C**) CRISPR-Cas9 plasmid delivery in the THP-1 cells. Confocal images showing the labeled plasmid in red (alone), the labeled plasmid in the presence of nuclei marker Hoechst 33342 in blue, the plasmid in the presence of the lysosomal marker (green) and the merged image displaying all the channels. White arrows pointed the plasmid signal overlapping with the nucleus.Data show representative images of the confocal studies in at least three independent experiments.

Similar nanoparticles loaded with RhB (i.e., ^GSDMD45^CRISPR-RhB-MSNs) were used to demonstrate the nanoparticle uptake by cells. The cellular uptake efficiency of the nanoparticles in the THP-1 monocytes was confirmed by flow cytometry (Figure 4A). The fluorescence confocal microscopy images showed remarkable RhB delivery in the THP-1 cells after 24 h of incubation with ^GSDMD45^CRISPR-RhB-MSNs (Figure 4B), which confirmed internalization and cargo release. We demonstrated proper CRISPR-Cas9 plasmid delivery in the THP-1 cells. For this purpose, the THP-1 cells were treated with the unloaded nanoparticles that contained the CRISPR-Cas9 plasmid labeled with the Cy5 dye (i.e., *^GSDMD45^CRISPR-MSNs) in the presence of a lysosomal marker. After 24 h, the confocal images demonstrated that the CRISPR-Cas9 plasmid was found in the cytoplasm, and no significant overlapping signals between lysosomes (green) and plasmid (red) were observed. Some plasmid signals overlapping the nucleus were noted (marked with the white arrows in Figure 4C). All these results suggest the proper internalization of CRISPR-MSNs into the THP-1 monocytes with the subsequent cargo and plasmid delivery.

**Figure 5:**
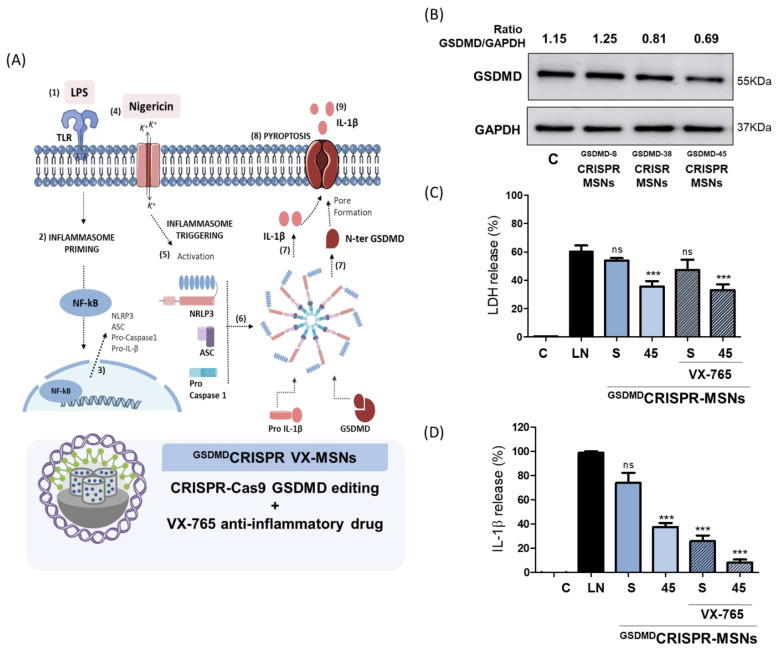
Dual one-shot anti-inflammatory therapy. (**A**) Scheme of the inflammatory response activation pathway by lipopolysaccharide (LPS) from *Escherichia coli* O111:B4 and nigericin (NG) exposure and the double-hit strategy design by using CRISPR-MSNs. (**B**) Western blot analysis of the GSDMD levels expressed in the cell lysates of the CRISPR-MSNs editing studies. Representative images of at least three independent experiments. (**C**) LDH release assay and (**D**) IL-1β levels. Data represent the mean ± SEM of at least three independent experiments. Statistical significance was determined by applying a one-way ANOVA analysis with multiple comparisons (*** *p* < 0.001). No significant differences were denoted as ns.

In order to evaluate whether MSNs could be used as a platform for dual anti-inflammatory therapy, the THP-1 cells were selected as a cellular model because they have been widely used for inflammatory disorder research, and it is a suitable in vitro cell model for studying immune modulation approaches [42]. In this case, the inflammatory response was induced by the stimulation with lipopolysaccharide (LPS) from *Escherichia coli* O111:B4 as the TLR4 ligand and potassium ionophore nigericin (NG) [43,44] (Figure 5A). After recognizing the first damage signals (Figure 5A) (1), such as LPS, different signaling pathways are triggered (2), and inflammasome components and cytokines are produced by the cell (3). Then NG induces intracellular damage (4) by triggering the activation of the NLPR3 inflammasome (5). NLPR3 oligomerizes and recruits ASC, which polymerizes into filaments to finally incorporate Caspase 1 by forming an inflammasome star-like structure (6) Caspase-1 is activated and cleaves pro-inflammatory cytokines, such as interleukin-1-beta (IL-1β), and gasdermin D (GSDMD), into their active forms (7). Active GSDMD (N-ter GSDMD) oligomerizes and forms pores in the cell membrane with subsequent cell lysis or pyroptosis (8) to allow the release of active interleukins (9). As a previous step, we evaluated proper GSDMD editing using the CRISPR-MSNs in the THP-1 cells by Western blot (Figure 5B). We observed that the ^GSDMD45^CRISPR-MSNs-treated cells revealed a significant decrease in GSDMD expression compared to ^GSDMD-S^CRISPR-MSNs and the untreated cells. These findings confirm that CRISPR-MSNs are a potential tool for CRISPR-Cas9 delivery and gene editing.

Finally, to confirm that the prepared nanoparticles were suitable for disease treatment by codelivering drugs and gene-editing machinery, the THP-1 cells were treated with ^GSDMD45^CRISPR-VX-MSNs and the unloaded ^GSDMD45^CRISPR-MSNs at 25 µg/mL, and the inflammatory response in cells was activated with LPS and NG. Similar nanoparticles containing a scrambled plasmid (i.e., ^GSDMD-S^CRISPR-VX-MSNs and ^GSDMD-S^CRISPR-MSNs) were also tested. Given that after the gene edition of GSDMD its protein expression diminished (Figure 5B), cell death by pyroptosis was quantified by measuring the release of enzyme lactate dehydrogenase (LDH), which is indicative of lytic programmed cell death (Figure 5C). Remarkable cell death was observed with the LPS + NG (LN) treatment, whereas a significant reduction in LDH release was found in the presence of ^GSDMD45^CRISPR-MSNs and ^GSDMD45^CRISPR-VX-MSNs, which was attributed to GSDMD editing. However, no changes in LDH release were observed upon the treatment of the THP-1 cells activated with LPS and NG (LN) with the nanoparticles containing a scrambled plasmid (i.e., ^GSDMD-S^CRISPR-MSNs and ^GSDMD-S^CRISPR-VX-MSNs).

In addition, the delivery of VX-765 from nanoparticles was expected to reduce the inflammatory response through caspase-1 inhibition and, thus, IL-1β reduction. The treatment of the THP-1 cells with the VX-765-loaded nanoparticles ^GSDMD-S^CRISPR-VX-MSNs significantly lowered the IL-1β levels (Figure 5D), which did not occur for ^GSDMD-S^CRISPR-MSNs. In contrast, IL-1β expression somewhat diminished with ^GSDMD45^CRISPR-MSNs, which was attributed to GSDMD editing and has been reported to regulate IL-1β secretion [45]. Moreover, only when the combined therapy was employed (GSDMD editing and VX-765 delivery) through the ^GSDMD45^CRISPR-VX-MSNs treatment, was greater inflammatory response reduction achieved. Distinct sgRNAs that target other GSDMD gene positions were also cloned, and the corresponding nanoparticles ^GSDMD38^CRISPR-MSNs and ^GSDMD38^CRISPR-VX-MSNs were prepared and tested in the THP-1 cells with similar results (Figure S6). All the above findings demonstrate a significant reduction in not only pyroptosis as a result of GSDMD editing, but also in inflammatory cytokines by VX-765 delivery when using ^GSDMD45^CRISPR-VX-MSNs. These results evidence the potential of MSNs as a scaffold to implement CRISPR-Cas9 and drug delivery combined therapies by using a single nanoparticle.

## 4. Discussion

Considerable attention has been paid to the potential of the gene-editing CRISPR/Cas9 technology, which has been quickly applied in the therapeutic field. For this purpose, the CRISPR/Cas9 system needs an effective and safe delivery carrier. In this respect, nanotechnology has led to the rapid improvement in applying this technology by providing powerful non-viral delivery tools [46,47]. Despite all efforts in recent years having been made to develop safer and more efficient nanocarriers to enhance CRISPR-Cas9 delivery, its combination with drug delivery and using, at the same time, a single nanocarrier is still an incipient concept in the therapeutic field with very few examples. The dual delivery of the CRISPR-Cas9 gene-editing system with a drug can be a promising approach to accurately handle complex diseases like cancer or inflammation. Here we envision that CRISPR-Cas9 delivery and drug release could be combined to enhance therapies in the inflammatory field. Although nanotechnology has centered on cancer therapy, and even by delivering the CRISPR-Cas9 gene-editing therapy, its application for managing inflammatory disorders is a less explored therapeutic field [48,49]. Accordingly, the versatility of MSNs has been previously demonstrated, which are a suitable nanocarrier to achieve dual drug delivery and CRISPR-Cas9 gene editing. Considering their big surface area and high loading capacity, MSNs can be loaded with large amounts of drugs. Besides, the external surface of MSNs can be easily functionalized with molecules to develop on-command drug release systems with either targeting ligands to enhance selectivity to cells and tissues or cationic groups to escape the endocytic pathway and allow DNA delivery [50,51,52].

Here we demonstrate that MSNs can efficiently codeliver a payload and CRISPR-Cas9 editing machinery in cells. In a first attempt, we evaluate this dual ability with the MSNs loaded with RhB as a drug model, capped with PEI, to achieve endosomal escape, and the CRISPR-Cas9 vector for the edition of GFP in cells. This nanodevice (^GFP38^CRISPR-RhB-MSNs) remains capped at a neutral physiological pH, whereas both the capping ensemble and cargo are delivered at an acidic pH inside cells. In addition, after CRISPR-Cas9 adsorption onto the PEI-coated MSNs, the stability of the plasmid is increased and protected from degradation. Then proper gene editing after the CRISPR-Cas9 release from MSNs is confirmed by monitoring the reduction of GFP intensity by achieving 30–50% editing efficacy. The obtained data are consistent with previous studies, in which organic nanoparticles have been used for CRISPR-Cas9 delivery, and report gene-editing efficacy results in the same order as this work does (20–40%) [53,54,55,56]. Apart from gene editing, we incorporate an advanced function by employing the same nanocarrier on-command drug delivery. The versatility of MSNs, the presence of the porous scaffold, and their unique properties all offer the possibility of proposing a dual gene and drug therapy to treat complex diseases.

Inflammation is a complex process in response to infectious agents, and is an injury that involves different mechanisms of induction, regulation, and resolution. When inflammation is uncontrolled and prevails with time, it can contribute to the development of diverse inflammatory disorders [57,58]. The insufficient ability to solve inflammation underlies a common basis of these inflammatory diseases produced by pathogens, endogenous signals, or tissue damage. An imbalance in the inflammatory cell population, and the increased presence of inflammatory mediators and chemokines, are the key factors responsible for the progression of uncontrolled inflammation and, thus, inflammatory diseases. Considering the role of elevated inflammatory cytokines, recent anti-inflammatory therapies mainly focus on lowering cytokines levels like IL-1β [59]. IL-1R antagonist anakinra and IL-1β antagonist canakinumab are FDA-approved and used in clinics [60,61]. In addition, inhibitors of caspase 1, such as VX-765, show significant inflammation inhibition by preventing IL-1β secretion, and are being tested in clinical trials [35,62]. However, the inhibition of downstream cytokines in the inflammatory pathway is not enough to completely deal with inflammation because cellular death triggered by inflammation (pyroptosis) is not inhibited. Indeed a few years ago, gasdermin D (GSDMD) was described as the key protein involved in the activation process of pyroptosis. GSDMD is cleaved to its active form (N-ter GSDMD), which oligomerizes and forms pores in the cell membrane with subsequent cell lysis, and death, and also allows the release of inflammatory interleukins. Recent studies have proved that after GSDMD gene deletion, recovery from inflammation is greater in murine models [36,63]. Considering these facts, we selected GSDMD as a target for gene editing to reduce its activity and to, therefore, diminish pyroptosis, whereas VX-765 delivery inhibits caspase-1 activity and pro-inflammatory cytokine release to improve the efficacy of current anti-inflammatory therapies.

In this case, the MSNs loaded with the VX-765 drug, coated with PEI and capped with a CRISPR-Cas9 plasmid to edit GSDMD were prepared (^GSDMD45^CRISPR-VX-MSNs). Our results show the significant reduction in pyroptosis by GSDMD editing and the inhibition of IL-1β release by VX-765 delivery. Regarding the combined effect as a result of the dual treatment, only when the combined therapy was employed (GSDMD editing and VX-765 delivery) was the reduction in the inflammatory response greater. This was attributed mainly to the reduction in pyroptosis, which also regulates the release of interleukin 1β. This is one of the few examples of nanoparticles capable of simultaneously delivering CRISPR-Cas9 gene-editing machinery and releasing drugs for one-shot treatments. The first use of MSNs as a dual platform for the gene-drug combined therapy was recently described for enhancing cancer therapy in hepatocellular carcinoma and melanoma [27,28]. As far as we know, these data are the first example of CRISPR-Cas9 editing machinery and drug codelivery by using a single nanoparticle to manage inflammatory disorders. We hope that these results open up new research opportunities in the inflammation field and confirm the potential of MSNs as a dual platform to deliver CRISPR-Cas9 machinery and drugs. Although CRISPR-Cas9 delivery by using MSNs is in an initial stage and challenges still remain, the versatility of this type of nanomaterial allows multiple combinations for gene editing by carrying plasmids, sgRNA, and Cas9 mRNA, or directly the Cas9 protein. Moreover, targeting ligands can be easily incorporated into MSNs to achieve targeted CRISPR-Cas9 delivery to thus reduce the off-target effects that derive from gene editing in non-targeted tissues [64]. Importantly as a dual platform, multiple combinations for gene editing and drug therapy can be envisioned by using MSNs by preparing advanced nanodevices to solve possible biomedical needs in complex diseases or restoring sensitivity in drug-resistant diseases like cancer or infections [64,65,66].

## 5. Conclusions

In summary, we report nanoparticles capable of efficiently codelivering CRISPR-Cas9 editing machinery and a payload in cells. Nanoparticles consist of MSNs loaded with RhB or the drug VX-765, and capped with PEI and the CRISPR-Cas9 vector, for the edition of GFP or GSDMD genes. Nanodevices remain capped at a neutral physiological pH, whereas both the capping ensemble and cargo are delivered at an acidic pH. The ^GFP38^CRISPR-RhB-MSNs in the U-2 OS-GFP cells lower GFP expression levels and codeliver RhB. Moreover, the inflammatory ^GSDMD45^CRISPR-VX-MSNs-treated THP-1 cells result in the delivery of the plasmid and the VX-765 drug with the consequent reduction in both cytokines and inflammatory cell death. To the best of our knowledge, these data represent one of the few examples of CRISPR-Cas9 editing machinery and cargo codelivery by using a single nanoparticle, and the first applied for the management of inflammatory disorders. Codelivering CRISPR-Cas9 editing machinery and a drug represents a potential tool for the one-shot treatment of diseases by combining different strategies with synergistic therapy or restoring sensitivity in drug-resistant malignancies.

## Data Availability

The data presented in this study are available on request from the corresponding author.

## Supplementary Materials

The following supporting information can be downloaded at: https://www.mdpi.com/article/10.3390/pharmaceutics14071495/s1. Figure S1. CRISPR vector design. Scheme S1. Nanoparticles nomenclature and composition. Figure S2. Nanoparticles standard characterization. Figure S3. Gene editing in U-2 OS-GFP cells. Figure S4. Gene editing and cargo co-delivery into U-2 OS-GFP cells. Scheme S2. Nanoparticles nomenclature and composition. Figure S5. Cell viability studies by WST-1 assays at different ^GSDMD45^CRISPR-MSNs concentrations at 24 (black bars) and 48 h (grey bars) in THP-1 cells. Figure S6. Gene editing and cargo co-delivery into THP-1 cells.

## References

[1] Mojica F.J.M., Montoliu L. (2016). On the Origin of CRISPR-Cas Technology: From Prokaryotes to Mammals. Trends Microbiol..

[2] Doudna J.A., Charpentier E. (2014). Genome Editing. The New Frontier of Genome Engineering with CRISPR-Cas9. Science.

[3] Mojica F.J., Ferrer C., Juez G., Rodríguez-Valera F. (1995). Long Stretches of Short Tandem Repeats Are Present in the Largest Replicons of the Archaea Haloferax Mediterranei and Haloferax Volcanii and Could Be Involved in Replicon Partitioning. Mol. Microbiol..

[4] Ahmar S., Mahmood T., Fiaz S., Mora-Poblete F., Shafique M.S., Chattha M.S., Jung K.-H. (2021). Advantage of Nanotechnology-Based Genome Editing System and Its Application in Crop Improvement. Front. Plant Sci..

[5] He Z.-Y., Men K., Qin Z., Yang Y., Xu T., Wei Y.-Q. (2017). Non-Viral and Viral Delivery Systems for CRISPR-Cas9 Technology in the Biomedical Field. Sci. China. Life Sci..

[6] Liu C., Zhang L., Liu H., Cheng K. (2017). Delivery Strategies of the CRISPR-Cas9 Gene-Editing System for Therapeutic Applications. J. Control. Release.

[7] Freen-van Heeren J.J., Popović B., Guislain A., Wolkers M.C. (2020). Human T Cells Employ Conserved AU-Rich Elements to Fine-Tune IFN-γ Production. Eur. J. Immunol..

[8] Schumann K., Lin S., Boyer E., Simeonov D.R., Subramaniam M., Gate R.E., Haliburton G.E., Ye C.J., Bluestone J.A., Doudna J.A. (2015). Generation of Knock-in Primary Human T Cells Using Cas9 Ribonucleoproteins. Proc. Natl. Acad. Sci. USA.

[9] Glass Z., Lee M., Li Y., Xu Q. (2018). Engineering the Delivery System for CRISPR-Based Genome Editing. Trends Biotechnol..

[10] Nelson C.E., Gersbach C.A. (2016). Engineering Delivery Vehicles for Genome Editing. Annu. Rev. Chem. Biomol. Eng..

[11] Zuris J.A., Thompson D.B., Shu Y., Guilinger J.P., Bessen J.L., Hu J.H., Maeder M.L., Joung J.K., Chen Z.-Y., Liu D.R. (2015). Cationic Lipid-Mediated Delivery of Proteins Enables Efficient Protein-Based Genome Editing In Vitro and In Vivo. Nat. Biotechnol..

[12] Miller J.B., Zhang S., Kos P., Xiong H., Zhou K., Perelman S.S., Zhu H., Siegwart D.J. (2017). Non-Viral CRISPR/Cas Gene Editing In Vitro and In Vivo Enabled by Synthetic Nanoparticle Co-Delivery of Cas9 MRNA and SgRNA. Angew. Chem. Int. Ed. Engl..

[13] Chen Z., Liu F., Chen Y., Liu J., Wang X., Chen A.T., Deng G., Zhang H., Liu J., Hong Z. (2017). Targeted Delivery of CRISPR/Cas9-Mediated Cancer Gene Therapy via Liposome-Templated Hydrogel Nanoparticles. Adv. Funct. Mater..

[14] Mout R., Ray M., Yesilbag Tonga G., Lee Y.-W., Tay T., Sasaki K., Rotello V.M. (2017). Direct Cytosolic Delivery of CRISPR/Cas9-Ribonucleoprotein for Efficient Gene Editing. ACS Nano.

[15] Yu K.-X., Qiao Z.-J., Song W.-L., Bi S. (2021). Correction to: DNA Nanotechnology for Multimodal Synergistic Theranostics. J. Anal. Test..

[16] Chen G., Abdeen A.A., Wang Y., Shahi P.K., Robertson S., Xie R., Suzuki M., Pattnaik B.R., Saha K., Gong S. (2019). A Biodegradable Nanocapsule Delivers a Cas9 Ribonucleoprotein Complex for In Vivo Genome Editing. Nat. Nanotechnol..

[17] Hamilton J.R., Tsuchida C.A., Nguyen D.N., Shy B.R., McGarrigle E.R., Sandoval Espinoza C.R., Carr D., Blaeschke F., Marson A., Doudna J.A. (2021). Targeted Delivery of CRISPR-Cas9 and Transgenes Enables Complex Immune Cell Engineering. Cell Rep..

[18] Wang P., Zhang L., Zheng W., Cong L., Guo Z., Xie Y., Wang L., Tang R., Feng Q., Hamada Y. (2018). Thermo-Triggered Release of CRISPR-Cas9 System by Lipid-Encapsulated Gold Nanoparticles for Tumor Therapy. Angew. Chem. Int. Ed. Engl..

[19] Noureddine A., Maestas-Olguin A., Saada E.A., LaBauve A.E., Agola J.O., Baty K.E., Howard T., Sabo J.K., Espinoza C.R.S., Doudna J.A. (2020). Engineering of Monosized Lipid-Coated Mesoporous Silica Nanoparticles for CRISPR Delivery. Acta Biomater..

[20] Alsaiari S.K., Patil S., Alyami M., Alamoudi K.O., Aleisa F.A., Merzaban J.S., Li M., Khashab N.M. (2018). Endosomal Escape and Delivery of CRISPR/Cas9 Genome Editing Machinery Enabled by Nanoscale Zeolitic Imidazolate Framework. J. Am. Chem. Soc..

[21] Hanahan D., Weinberg R.A. (2011). Hallmarks of Cancer: The next Generation. Cell.

[22] Liu Q., Zhang T.-X., Zheng Y., Wang C., Kang Z., Zhao Y., Chai J., Li H.-B., Guo D.-S., Liu Y. (2021). Calixarene-Embedded Nanoparticles for Interference-Free Gene–Drug Combination Cancer Therapy. Small.

[23] Nastiuk K.L., Krolewski J.J. (2016). Opportunities and Challenges in Combination Gene Cancer Therapy. Adv. Drug Deliv. Rev..

[24] Argyo C., Weiss V., Bräuchle C., Bein T. (2014). Multifunctional Mesoporous Silica Nanoparticles as a Universal Platform for Drug Delivery. Chem. Mater..

[25] García-Fernández A., Aznar E., Martínez-Máñez R., Sancenón F. (2020). New Advances in In Vivo Applications of Gated Mesoporous Silica as Drug Delivery Nanocarriers. Small.

[26] Aznar E., Oroval M., Pascual L., Murguía J.R., Martínez-Máñez R., Sancenón F. (2016). Gated Materials for On-Command Release of Guest Molecules. Chem. Rev..

[27] Zhang B.-C., Luo B.-Y., Zou J.-J., Wu P.-Y., Jiang J.-L., Le J.-Q., Zhao R.-R., Chen L., Shao J.-W. (2020). Co-Delivery of Sorafenib and CRISPR/Cas9 Based on Targeted Core–Shell Hollow Mesoporous Organosilica Nanoparticles for Synergistic HCC Therapy. ACS Appl. Mater. Interfaces.

[28] Liu Q., Wang C., Zheng Y., Zhao Y., Wang Y., Hao J., Zhao X., Yi K., Shi L., Kang C. (2020). Virus-like Nanoparticle as a Co-Delivery System to Enhance Efficacy of CRISPR/Cas9-Based Cancer Immunotherapy. Biomaterials.

[29] Medzhitov R. (2010). Inflammation 2010: New Adventures of an Old Flame. Cell.

[30] Libby P. (2007). Inflammatory Mechanisms: The Molecular Basis of Inflammation and Disease. Nutr. Rev..

[31] Perretti M., Leroy X., Bland E.J., Montero-Melendez T. (2015). Resolution Pharmacology: Opportunities for Therapeutic Innovation in Inflammation. Trends Pharmacol. Sci..

[32] Díaz-González F., Sánchez-Madrid F. (2015). NSAIDs: Learning New Tricks from Old Drugs. Eur. J. Immunol..

[33] Cain D.W., Cidlowski J.A. (2017). Immune Regulation by Glucocorticoids. Nat. Rev. Immunol..

[34] Platnich J.M., Muruve D.A. (2019). NOD-like Receptors and Inflammasomes: A Review of Their Canonical and Non-Canonical Signaling Pathways. Arch. Biochem. Biophys..

[35] Wannamaker W., Davies R., Namchuk M., Pollard J., Ford P., Ku G., Decker C., Charifson P., Weber P., Germann U.A. (2007). (S)-1-((S)-2-{[1-(4-Amino-3-Chloro-Phenyl)-Methanoyl]-Amino}-3,3-Dimethyl-Butanoyl)-Pyrrolidine-2-Carboxylic Acid ((2R,3S)-2-Ethoxy-5-Oxo-Tetrahydro-Furan-3-Yl)-Amide (VX-765), an Orally Available Selective Interleukin (IL)-Converting Enzyme/Caspase-1 In. J. Pharmacol. Exp. Ther..

[36] Liu X., Zhang Z., Ruan J., Pan Y., Magupalli V.G., Wu H., Lieberman J. (2016). Inflammasome-Activated Gasdermin D Causes Pyroptosis by Forming Membrane Pores. Nature.

[37] Fujihara Y., Ikawa M. (2014). CRISPR/Cas9-Based Genome Editing in Mice by Single Plasmid Injection. Methods Enzymol..

[38] Graham D.B., Root D.E. (2015). Resources for the Design of CRISPR Gene Editing Experiments. Genome Biol..

[39] Conner S.D., Schmid S.L. (2003). Regulated Portals of Entry into the Cell. Nature.

[40] Lu F., Wu S.-H., Hung Y., Mou C.-Y. (2009). Size Effect on Cell Uptake in Well-Suspended, Uniform Mesoporous Silica Nanoparticles. Small.

[41] Kumari A., Yadav S.K. (2011). Cellular Interactions of Therapeutically Delivered Nanoparticles. Expert Opin. Drug Deliv..

[42] Chanput W., Mes J.J., Wichers H.J. (2014). THP-1 Cell Line: An in Vitro Cell Model for Immune Modulation Approach. Int. Immunopharmacol..

[43] Pétrilli V., Papin S., Dostert C., Mayor A., Martinon F., Tschopp J. (2007). Activation of the NALP3 Inflammasome Is Triggered by Low Intracellular Potassium Concentration. Cell Death Differ..

[44] Franchi L., Muñoz-Planillo R., Núñez G. (2012). Sensing and Reacting to Microbes through the Inflammasomes. Nat. Immunol..

[45] Evavold C.L., Ruan J., Tan Y., Xia S., Wu H., Kagan J.C. (2018). The Pore-Forming Protein Gasdermin D Regulates Interleukin-1 Secretion from Living Macrophages. Immunity.

[46] Saw P.E., Cui G.-H., Xu X. (2022). Nanoparticles-Mediated CRISPR/Cas Gene Editing Delivery System. ChemMedChem.

[47] Taha E.A., Lee J., Hotta A. (2022). Delivery of CRISPR-Cas Tools for in Vivo Genome Editing Therapy: Trends and Challenges. J. Control. Release.

[48] Alshamrani M. (2022). Broad-Spectrum Theranostics and Biomedical Application of Functionalized Nanomaterials. Polymers.

[49] Tagami T., Ozeki T. (2017). Recent Trends in Clinical Trials Related to Carrier-Based Drugs. J. Pharm. Sci..

[50] Saw P.E., Cui G.-H., Xu X., Taha E.A., Lee J., Hotta A., Alshamrani M., Tagami T., Ozeki T., Gisbert-Garzarán M. (2022). An Update on Mesoporous Silica Nanoparticle Applications in Nanomedicine. Pharmaceutics.

[51] Gisbert-Garzarán M., Lozano D., Vallet-Regí M. (2020). Mesoporous Silica Nanoparticles for Targeting Subcellular Organelles. Int. J. Mol. Sci..

[52] Gao Y., Gao D., Shen J., Wang Q. (2020). A Review of Mesoporous Silica Nanoparticle Delivery Systems in Chemo-Based Combination Cancer Therapies. Front. Chem..

[53] Qiao J., Sun W., Lin S., Jin R., Ma L., Liu Y. (2019). Cytosolic Delivery of CRISPR/Cas9 Ribonucleoproteins for Genome Editing Using Chitosan-Coated Red Fluorescent Protein. Chem. Commun..

[54] Liu J., Chang J., Jiang Y., Meng X., Sun T., Mao L., Xu Q., Wang M. (2019). Fast and Efficient CRISPR/Cas9 Genome Editing In Vivo Enabled by Bioreducible Lipid and Messenger RNA Nanoparticles. Adv. Mater..

[55] Hosseini E.S., Nikkhah M., Hosseinkhani S. (2019). Cholesterol-Rich Lipid-Mediated Nanoparticles Boost of Transfection Efficiency, Utilized for Gene Editing by CRISPR-Cas9. Int. J. Nanomed..

[56] Sun D., Sun Z., Jiang H., Vaidya A.M., Xin R., Ayat N.R., Schilb A.L., Qiao P.L., Han Z., Naderi A. (2019). Synthesis and Evaluation of PH-Sensitive Multifunctional Lipids for Efficient Delivery of CRISPR/Cas9 in Gene Editing. Bioconjug. Chem..

[57] Chen L., Deng H., Cui H., Fang J., Zuo Z., Deng J., Li Y., Wang X., Zhao L. (2018). Inflammatory Responses and Inflammation-Associated Diseases in Organs. Oncotarget.

[58] Medzhitov R. (2008). Origin and Physiological Roles of Inflammation. Nature.

[59] Tabas I., Glass C.K. (2013). Anti-Inflammatory Therapy in Chronic Disease: Challenges and Opportunities. Science.

[60] Ramírez J., Cañete J.D. (2018). Anakinra for the Treatment of Rheumatoid Arthritis: A Safety Evaluation. Expert Opin. Drug Saf..

[61] Rondeau J.-M., Ramage P., Zurini M., Gram H. (2015). The Molecular Mode of Action and Species Specificity of Canakinumab, a Human Monoclonal Antibody Neutralizing IL-1β. MAbs.

[62] Yang Y., Wang H., Kouadir M., Song H., Shi F. (2019). Recent Advances in the Mechanisms of NLRP3 Inflammasome Activation and Its Inhibitors. Cell Death Dis..

[63] Silva C.M.S., Wanderley C.W.S., Veras F.P., Sonego F., Nascimento D.C., Gonçalves A.V., Martins T.V., Cólon D.F., Borges V.F., Brauer V.S. (2021). Gasdermin D Inhibition Prevents Multiple Organ Dysfunction during Sepsis by Blocking NET Formation. Blood.

[64] Wang S.-W., Gao C., Zheng Y.-M., Yi L., Lu J.-C., Huang X.-Y., Cai J.-B., Zhang P.-F., Cui Y.-H., Ke A.-W. (2022). Current Applications and Future Perspective of CRISPR/Cas9 Gene Editing in Cancer. Mol. Cancer.

[65] Dubey A.K., Kumar Gupta V., Kujawska M., Orive G., Kim N.-Y., Li C.-Z., Kumar Mishra Y., Kaushik A. (2022). Exploring Nano-Enabled CRISPR-Cas-Powered Strategies for Efficient Diagnostics and Treatment of Infectious Diseases. J. Nanostruct. Chem..

[66] Chandrasekaran A.P., Karapurkar J.K., Chung H.Y., Ramakrishna S. (2022). The Role of the CRISPR-Cas System in Cancer Drug Development: Mechanisms of Action and Therapy. Biotechnol. J..

