# Supplementary material for: Nanodevices for the Efficient Codelivery of CRISPR-Cas9 Editing Machinery and an Entrapped Cargo: A Proposal for Dual Anti-Inflammatory Therapy
Source: Pharmaceutics. 2022 Jul 19;14(7):1495. doi: 10.3390/pharmaceutics14071495 (PMC9322049; doi:10.3390/pharmaceutics14071495)
Supplement: Supplementary file 1 [file pharmaceutics-14-01495-s001.zip › pharmaceutics-1802428-supplementary.pdf]

pharmaceutics

MDPI

Supplementary Materials: Nanodevices for the Efficient Codelivery of CRISPR-Cas9 Editing Machinery and an Entrapped Cargo. A Proposal for Dual Anti-inflammatory Therapy.

Alba García-Fernández, Gema Vivo-Llorca, Mónica Sancho, Alicia Belén García-Jareño, Laura Ramírez-Jiménez, Eloísa Barber-Cano, José Ramón Murguía, Mar Orzáez, Félix Sancenón and Ramón Martínez-Máñez

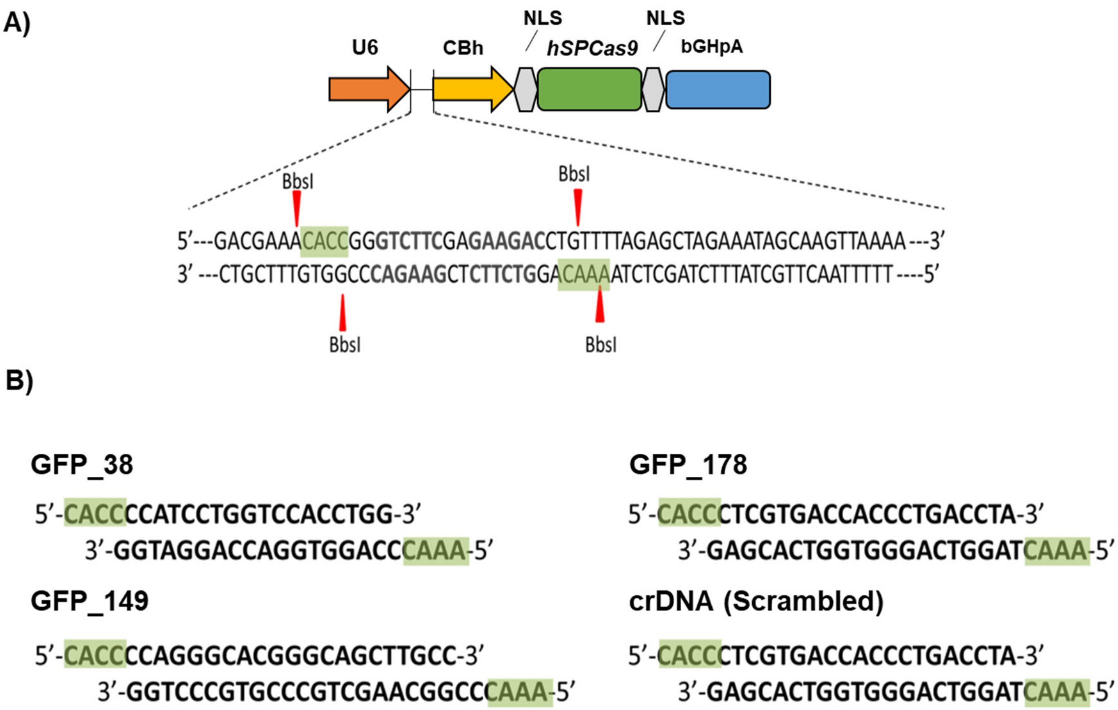

Figure S1. CRISPR vector design. A) Map image of pX330-U6-Chimeric_BB-CBh-hSpCas9 and the schematic representation of the guide sequence insertion site. This plasmid contains two expression cassettes, a human codon optimized SpCas9 or SpCas9n, and the single guide RNA. The vector can be digested using BbsI, and a pair of annealed oligos can be cloned into the vector. B) Guide sequences selected to edit the expression of the GFP gene at positions 38, 149, and 178. A scrambled crDNA was designed as a guide sequence that does not match with GFP, as a control of editing.

Nanoparticles nomenclature	Vector	Gate	Cargo	Support	Scheme
**MSNs**				MSNs	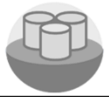
**PEI-MSNs**		PEI		MSNs	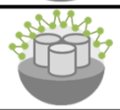
**PEI-RhB-MSNs**		PEI	RhB	MSNs	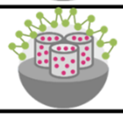
**^{scrambled}CRISPR-MSNs**	^{scrambled}CRISPR	PEI		MSNs	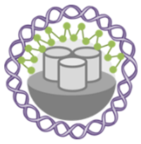
**$^{\text{GFP38}}$CRISPR-MSNs**	$^{\text{GFP38}}$CRISPR	PEI		MSNs	
**$^{\text{GFP149}}$CRISPR-MSNs**	$^{\text{GFP149}}$CRISPR	PEI		MSNs	
**$^{\text{GFP178}}$CRISPR-MSNs**	$^{\text{GFP178}}$CRISPR	PEI		MSNs	
**$^{\text{GFP38}}$CRISPR-RhB*-MSNs**	$^{\text{GFP38}}$CRISPR	PEI	RhB*	MSNs	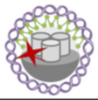
**^{scrambled}CRISPR-RhB-MSNs**	^{scrambled}CRISPR	PEI	RhB	MSNs	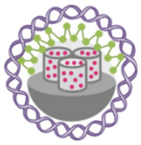
**$^{\text{GFP38}}$CRISPR-RhB-MSNs**	$^{\text{GFP38}}$CRISPR	PEI	RhB	MSNs	
**$^{\text{GFP149}}$CRISPR-RhB-MSNs**	$^{\text{GFP149}}$CRISPR	PEI	RhB	MSNs	
**$^{\text{GFP178}}$CRISPR-RhB-MSNs**	$^{\text{GFP178}}$CRISPR	PEI	RhB	MSNs	

Scheme S1. Nanoparticles nomenclature and composition. RhB indicated that dye was loaded in the inner of the porous network of the inorganic scaffold whereas RhB* indicated that dye was covalently anchored onto the support.

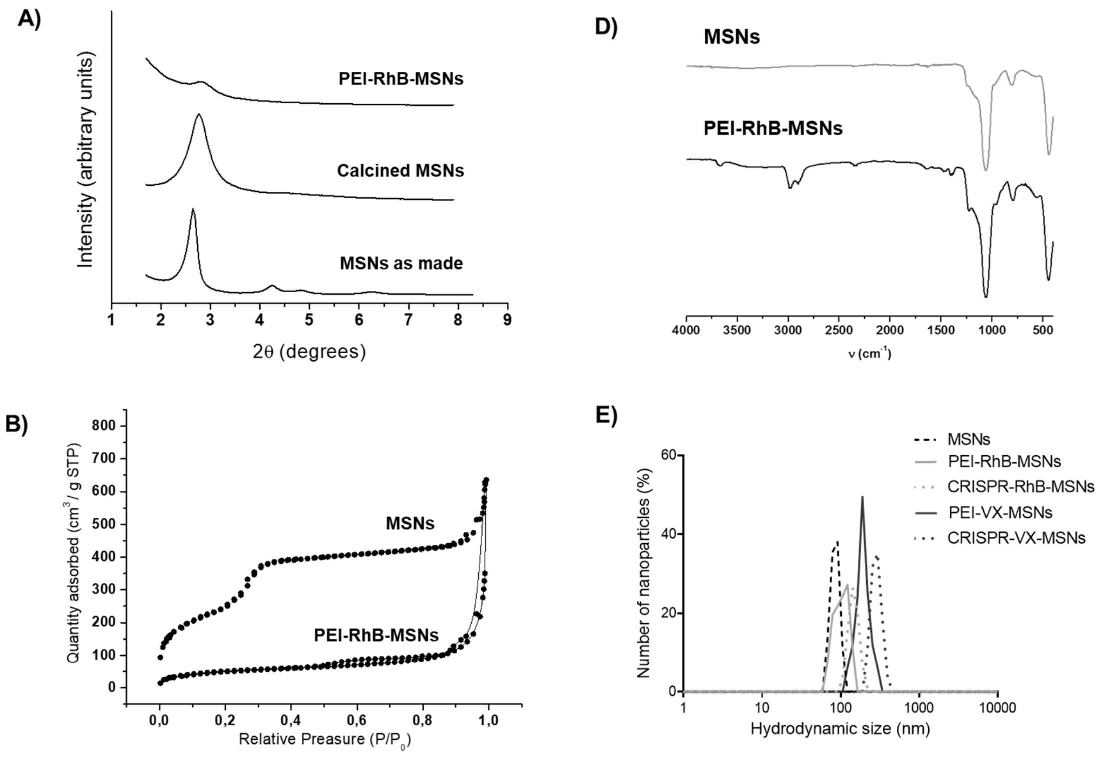

C)

Solid	S_{BET} ($m^2 g^{-1}$)	Pore volume ($cm^3 g^{-1}$)	Pore size (nm)
**MSNs**	1166.59	0.89	2.4
**PEI-RhB-MSNs**	172.08	0.14	-

Figure S2. Nanoparticles standard characterization**.** A) Powder X-ray patterns of MSNs as made, calcined MSNs, and PEI-RhB-MSNs. The characteristic (100) diffraction peak was observed indicating the preservation of the mesoporous structure after the functionalization processes. B) Nitrogen adsorption-desorption isotherm for MSNs and PEI-RhB-MSNs. The isotherm for the starting MSNs corresponds to a type IV isotherm, typical of these materials. In contrast, the isotherm obtained for PEI-RhB-MSNs is typical of mesoporous materials with filled mesopores with a marked decrease in the specific surface when compared with MSNs. C) S_{BET}, pore volume, and pore size for starting MSNs and PEI-RhB-MSNs obtained from N_2 adsorption-desorption isotherm studies. D) FTIR spectra of PEI-RhB-MSNs showing the symmetric and asymmetric stretching bands of amine moieties from PEI in the 3100-2900 cm^{-1} range indicating the proper PEI-coating of the nanoparticles. E) Hydrodynamic size of MSNs, PEI-RhB-MSNs, PEI-VX-MSNs, ^{GFP38}CRISPR-RhB-MSNs, and ^{GSDMD45}CRISPR-VX-MSNs.

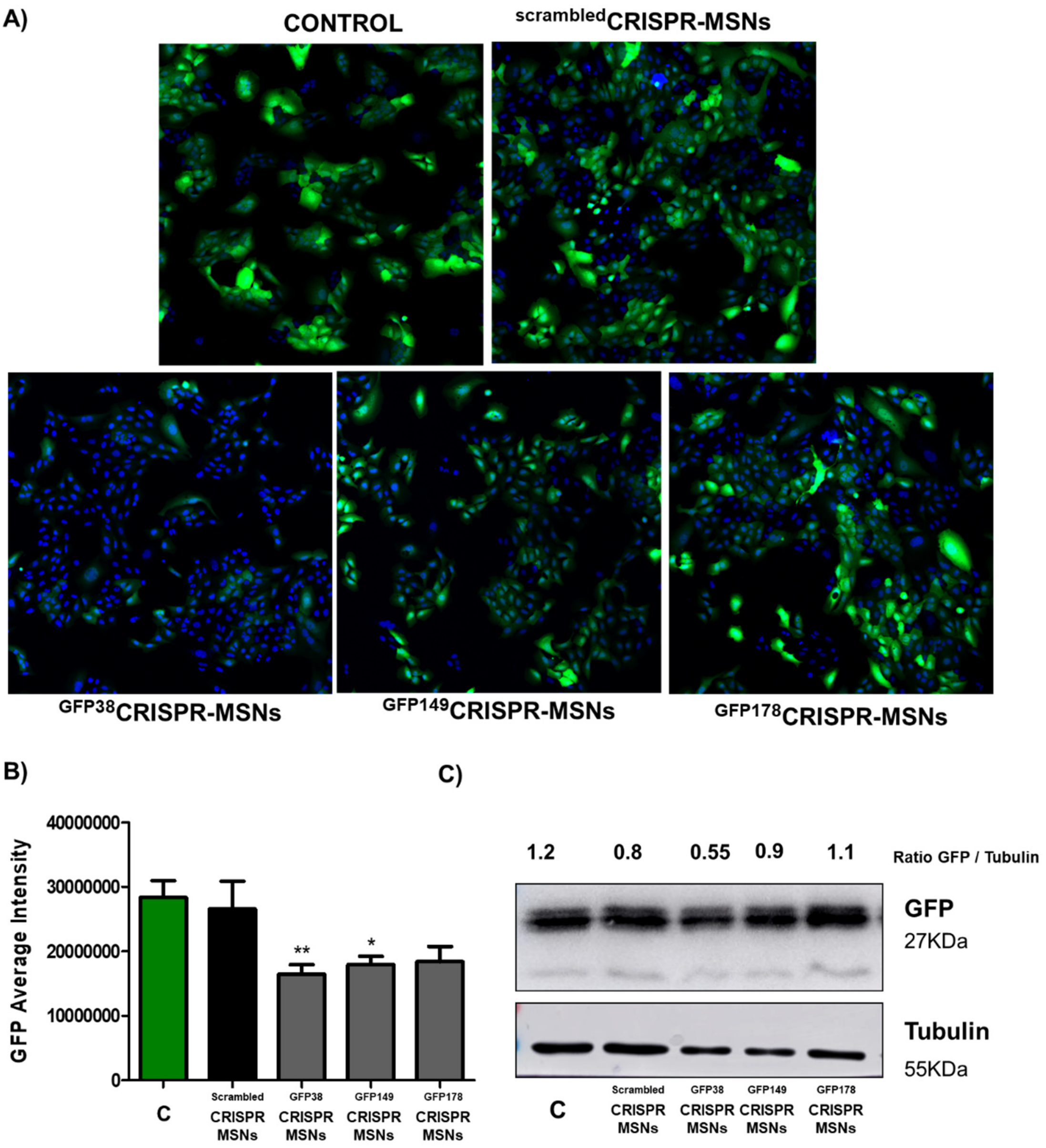

Figure S3. Gene editing in U-2 OS-GFP cells. A) Confocal microscopy images of genome editing by CRISPR-Cas9 system delivered by MSNs as nanocarriers. The transfection efficiency is judged by the fluorescent intensity and the proportion of cells in the population showing GFP expression. In green GFP cells and blue marked the nucleus with Hoechst 4332. B) GFP quantification by confocal image analysis. C) Western blot analysis of GFP levels expressed in cell lysates of CRISPR-MSNs editing studies. Data represent the mean ± SEM of at least three independent experiments (* $p < 0.05$, ** $p < 0.025$, *** $p < 0.001$).

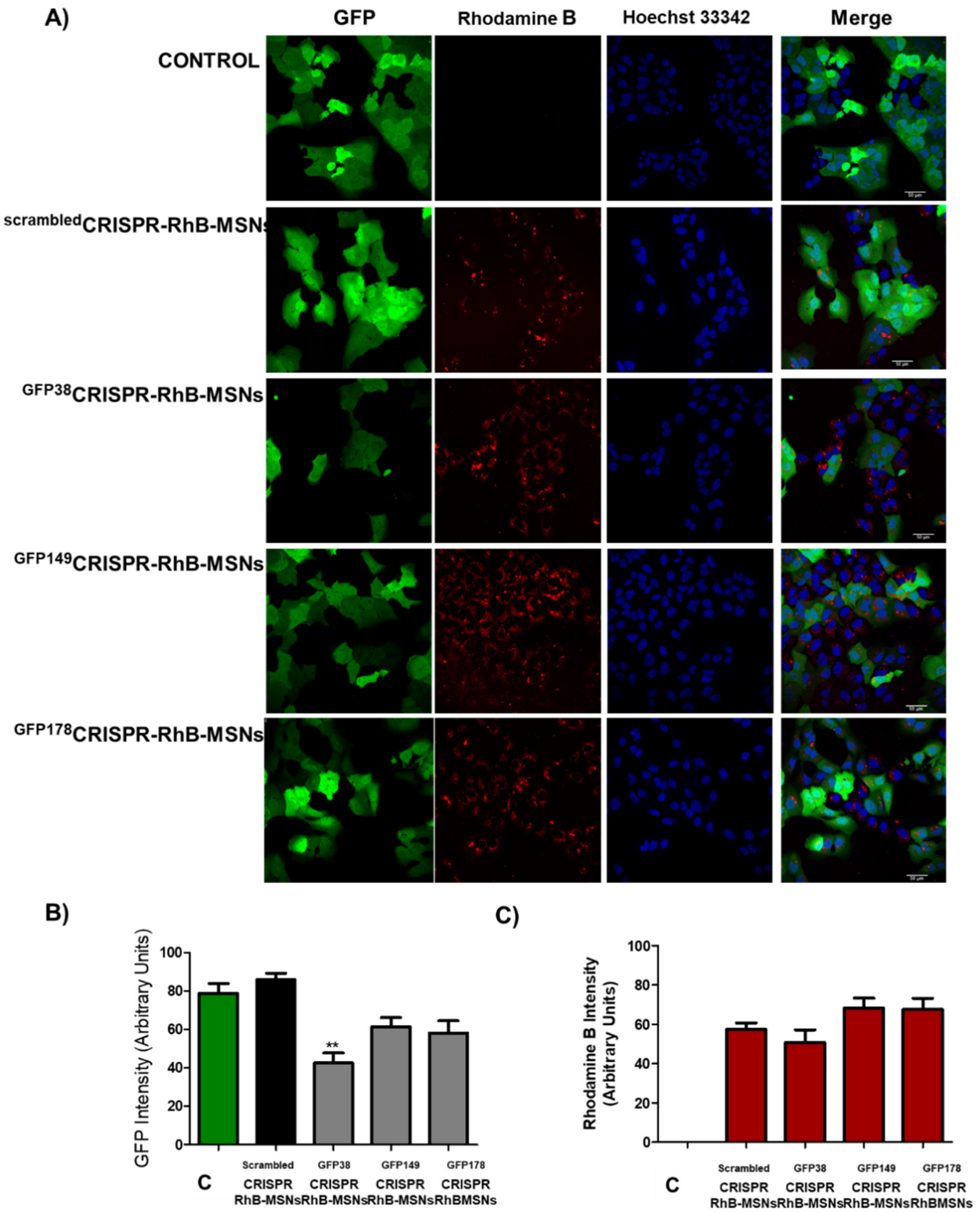

Figure S4. Gene editing and cargo co-delivery into U-2 OS-GFP cells. A) Confocal microscopy images of genome editing and cargo delivery from CRISPR-RhB-MSNs. The transfection efficiency is judged by the fluorescent intensity and proportion of cells in the population showing GFP (green) expression and the delivery efficiency by the fluorescent intensity of rhodamine B (red) and nucleus in blue marked with Hoechst 4332. B) Quantification of GFP intensity by confocal images analysis. The green bar represents untreated cells as negative control (C). C) Quantification of rhodamine B intensity delivered from nanoparticles by confocal images analysis. Untreated cells are referred to as C, as a negative control, in which rhodamine B-associated fluorescence is not detected, and normalized data results in 0%. Data represent the mean ± SEM of at least three independent experiments (* $p < 0.05$, ** $p < 0.025$, *** $p < 0.001$).

Nanoparticles nomenclature	Vector	Gate	Cargo	Support	Scheme
**MSNs**				MSNs	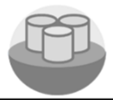
**PEI-MSNs**		PEI		MSNs	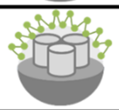
**PEI-RhB-MSNs**		PEI	RhB	MSNs	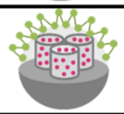
**PEI-VX-MSNs**		PEI	VX-765	MSNs	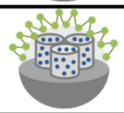
**$^{\text{GSDMD-S}}$CRISPR-MSNs**	$^{\text{GSDMD-S}}$CRISPR	PEI		MSNs	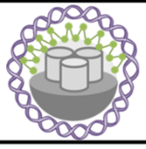
**$^{\text{GSDMD38}}$CRISPR-MSNs**	$^{\text{GSDMD37}}$CRISPR	PEI		MSNs	
**$^{\text{GSDMD45}}$CRISPR-MSNs**	$^{\text{GSDMD45}}$CRISPR	PEI		MSNs	
**$^{\text{*GSDMD45}}$CRISPR-MSNs**	$^{\text{GSDMD45}}$CRISPR Cy5 labelled	PEI		MSNs	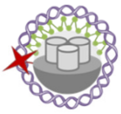
**$^{\text{GSDMD45}}$CRISPR-RhB-MSNs**	$^{\text{GSDMD45}}$CRISPR	PEI	RhB	MSNs	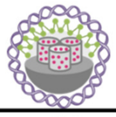
**$^{\text{GSDMD-S}}$CRISPR-VX-MSNs**	$^{\text{GSDMD-S}}$CRISPR	PEI	VX-765	MSNs	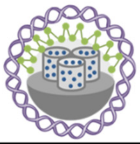
**$^{\text{GSDMD38}}$CRISPR-VX-MSNs**	$^{\text{GSDMD37}}$CRISPR	PEI	VX-765	MSNs	
**$^{\text{GSDMD45}}$CRISPR-VX-MSNs**	$^{\text{GSDMD45}}$CRISPR	PEI	VX-765	MSNs	

Scheme S2. Nanoparticles nomenclature and composition.

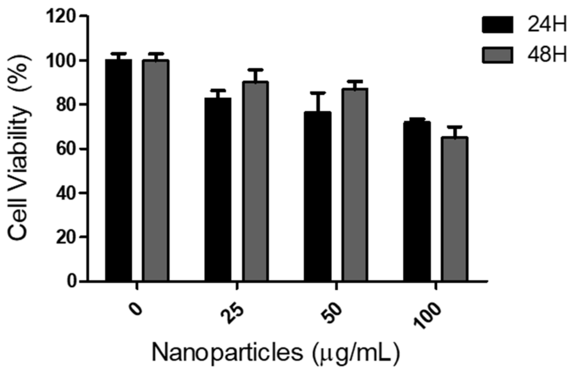

Figure S5. Cell viability studies by WST-1 assays at different $^{\text{GSDMD45}}$CRISPR-MSNs concentrations at 24 (black bars) and 48 hours (grey bars) in THP-1 cells.

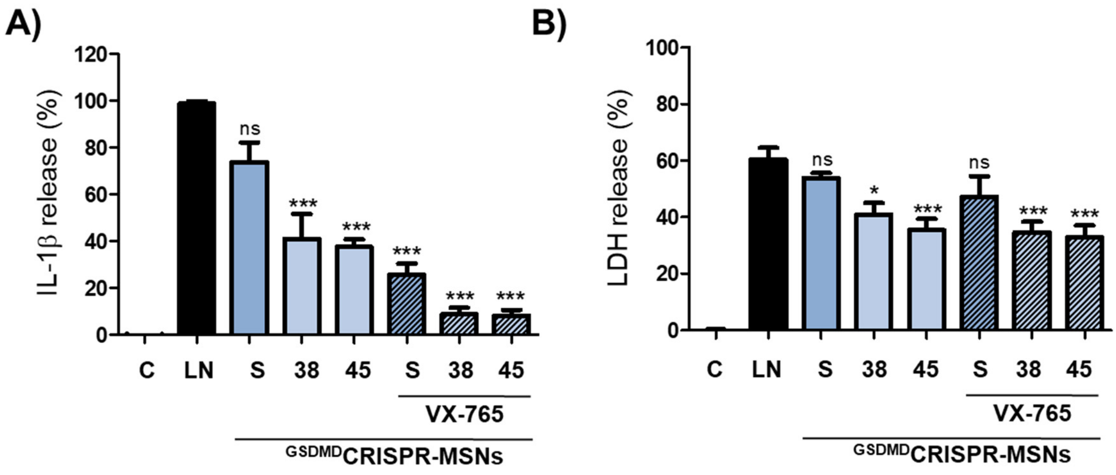

Figure S6. Gene editing and cargo co-delivery into THP-1 cells. A) LDH release assay and B) IL-1β levels. Data represent the mean ± SEM of at least three independent experiments (* $p < 0.05$, ** $p < 0.025$, *** $p < 0.001$).
